# Causal Model Analysis of the Effect of Formalism, Fear of Infection, COVID-19 Stress on Firefighters’ Post-Traumatic Stress Syndrome and Insomnia

**DOI:** 10.3390/ijerph20021097

**Published:** 2023-01-08

**Authors:** Yun-Ming Tang, Tsung-Lin Wu, Hsiang-Te Liu

**Affiliations:** 1 Asia Pacific Society of Fire Engineering, Kaohsiung City 825, Taiwan; 2 Department of Leisure Management, I-Shou University, Kaohsiung City 84001, Taiwan; 3 Department of Public Affairs and Administration, Ming Chuan University, Taoyuan City 333, Taiwan

**Keywords:** formalism, fear of family infection, COVID-19 stress, post-traumatic stress disorder, problem-focused strategies, insomnia

## Abstract

As the front line of epidemic prevention, firefighters are responsible for the transportation of infected cases. Firefighters are under a lot of stress from the new COVID-19, especially the fear that they may contract the virus at work and spread the virus to their families. In particular, the framework of this study incorporates Riggs’ formalism variables. When firefighters think that the epidemic prevention regulations are inconsistent with the actual epidemic prevention, it will increase their work pressure on COVID-19. In this study, firefighters from all over Taiwan were used as the respondents, and a total of 453 respondents were obtained. This study uses confirmatory factor analysis and structural equation modeling to test the established hypotheses. The findings confirm that formalism, fear of self and family infection are positively influencing COVID-19 stress. COVID-19 stress positively affects PTSD and insomnia. COVID-19 stress negatively affects problem-focused strategies. Problem-focused strategies negatively affect post-traumatic stress disorder.

## 1. Introduction

PSTD (post-traumatic stress disorder) is a mental disorder that is triggered by experiencing traumatic events such as earthquakes, typhoons, and SARS [1], or witnessing death and injury in tragic events [2]. There are also some studies confirming that a high degree of psychological stress can cause PSTD [3,4]. It has been evidenced by a number of studies that the COVID-19 pandemic can trigger and worsen post-traumatic stress disorder [5]. COVID-19 stress can be defined as a change in work that causes physical, emotional, or psychological strain caused by COVID-19 [5]. Lahav found that people who had previously experienced traumatic events were more likely to develop PTSD during the COVID-19 pandemic [6].

COVID-19 has become a global stressor because it is difficult to predict and control [5]. Kira et al. adopted the concept of psychological stress proposed by Lazarus and Folkman to measure the stress caused by COVID-19 [7,8]. The stresses triggered by COVID-19 include anxiety, nervousness, and fear. Studies have pointed out that COVID-19 is a special type of trauma in which traumatic stress persists [9]. The traumatic stress of COVID-19 lasts for a long time, which reduces individuals’ coping capabilities against stress [6]. The traumatic stress of COVID-19 comes from the fear of an unknown virus and the fear of family members being infected [9].

Many studies conducted recently have indicated that COVID-19 can cause an impact on front-line workers’ mental health [10,11]. Other studies have also confirmed the correlation between the COVID-19 pandemic and anxiety, depression, exhaustion, and stress [12,13]. Ahorsu et al. point out that the pain of COVID-19 creates fear and depression among front-line workers, which affects their work performance and mental health [14]. In particular, studies have pointed out that front-line healthcare workers often encounter patients who suffer or die as a result of COVID-19, and this makes the front-line workers more prone to post-traumatic stress [15].

Whether individuals have the ability to regulate emotions to mitigate emotional stress is an important topic in sleep physiology [16]. Stress and anxiety are causes of insomnia [17,18]. Mental problems are thought to be associated with insomnia [19,20]. A study by Salari et al. points out that stress is the main cause of insomnia among medical workers during the pandemic. Sleep disruption caused by stress is called insomnia [21,22]. Insomnia is considered a sleep disorder that can make it hard for people to fall asleep or make them wake up too early, affecting their daytime mental condition [23]. During the pandemic, internet searches for insomnia increased, suggesting that the pandemic has triggered the problem of insomnia [24]. Insomnia is considered to be a symptom that follows stress and anxiety [20,25].

Fear is the warning signal of individuals triggered by a danger or threat in the environment, urging them to change their behavior in order to adapt to the environment [26]. In the early stages of the Ebola and MERS pandemics, people were vaccinated due to fear [27]. High levels of fear can lead to psychological burdens, including anxiety, depression, post-traumatic stress disorder, stress, etc. [26,28,29].

When the level of fear increases, many psychological changes will take place [30,31]. Fear of COVID-19 can even lead to post-traumatic stress disorder [32]. In the early stages of the COVID-19 pandemic, fear of infection with COVID-19 received significant attention for research [33]. Many studies have also confirmed that the fear of being infected by COVID-19 can cause stress and anxiety [14,34]. For everyone, fear is a self-protection mechanism [35]. However, psychological fear may also lead to post-traumatic stress disorder [36]. Fear of being infected with COVID-19 also creates stress for many students [34].

Some researchers have pointed out that Riggs regards American society as the standard of a diffracted society, which has been discussed and questioned [37]. Taking the performance evaluation of civil servants in Taiwan as an example, every year, 75% of civil servants receive a grade of A without an actual performance evaluation, while the rest of the civil servants take turns to receive a grade of B, making performance evaluations formalistic [38]. Some researchers believe that prismatic societies do not only exist in low-development societies, but also in societies with various levels of development [37].

The formalism proposed by Riggs is characterized by ritualistic methods, lack of authorization, and centralization. The difference between legal norms and effective implementation also creates a gap between norms and realities [39]. Civil servants who do not have standards for administrative performance will not implement them pragmatically [39]. During the pandemic, if firefighters do not receive the required authorization in dealing with emergencies, their stress resulting from COVID-19 will increase. It is difficult for developing countries to implement organic-model organization [40]. The relatively flexible design of organic model organization will bring some anxiety to the supervisor [41]. This is the reason why administrations in developing countries have difficulty establishing organic model organization, and it is more difficult for mechanistic model organization to deal with the COVID-19 pandemic. There are many informal administrative behaviors in developing countries that hinder the achievement of organizational goals [39,42]. This study mainly explores whether firefighters’ personal and family fear of infection affects their work stress. Does the work stress of firefighters increase PTSD and insomnia problems? Do firefighters’ strategies for coping with COVID-19 stress reduce PTSD? This study is the first to examine whether policy formalism increases firefighters’ perceived job stress.

## 2. Literature Review and Hypotheses Development

The stress of COVID-19 comes from the fear of future infection and death [43,44], as well as economic hardship and changes in daily life [33,45]. In addition to worries about the infection of family members and friends, there are also uncertainties about the future development of the pandemic. From the examples of SARS, MERS, and Ebola in the past, it is believed that they will create PTSD and anxiety [46].

The unpredictability and uncertainty caused by COVID-19, as well as social isolation policies, have put stress on ordinary people [47]. Demertzis and Eyerman point out that changes in daily life, loss of trust in supervisors, and negative perception on the media are all signs of post-traumatic stress disorder [48]. The pandemic has enabled individuals to experience life-threatening situations and witness the loss of loved ones, which are regarded as stressful and traumatic events [49]. Studies conducted by militaries in the past have confirmed that the deeper the life-threatening feeling perceived by an individual, the higher the probability of the individual to suffer from post-traumatic stress disorder.

Many studies conducted recently have shown that the COVID-19 pandemic will lead to PTSD [50,51]. A survey in Italy also found that PTSD symptoms were up almost 30% [52]. With the spread of COVID-19, healthcare workers experienced symptoms such as irritability, difficulty in managing emotions, and feelings of stress [53]. Some past studies have discovered that front-line workers are prone to PTSD [54]. Firefighters are front-line workers for transporting COVID-19 patients and people who need to quarantine. They face considerable COVID-19 stress and are prone to PTSD.

**H1.**  *COVID-19 stress positively affects PTSD.*

When perceived stress increases, the chance of insomnia increases. “Insomnia” is defined as a condition of being unable to fall asleep easily, or stay asleep for more than 7 h [55]. Stress and depression develop when people are exposed to stressors over a long period of time [56]. The stress perceived by individuals can be used to evaluate threat. When stress increases, their body, emotion, and mind will be negatively affected [57,58].

Past studies have shown that the psychological stress of front-line workers during the pandemic is often higher than that of other occupations [59]. Psychological stress and trauma are both causes of insomnia for front-line workers. Taking the SARS outbreak that occurred in the past as an example, the healthcare workers who dealt with SARS on the front line experienced the problem of poor sleeping quality [60]. The front-line police officers responsible for implementing quarantine control, distributing epidemic prevention supplies, and carrying out curfew orders are not only busy with their work, but also are exposed to the high risk of contact with COVID-19 patients. They must have experienced great mental stress and suffered from insomnia. Since the outbreak of COVID-19, studies have also shown that front-line workers have suffered from insomnia [61]. Firefighters experiencing the stress of COVID-19 are prone to insomnia.

**H2.**  *COVID-19 stress positively affects insomnia.*

Past studies have shown that medical school students mainly adopt problem-focused strategies in the face of the stress derived from the COVID-19 pandemic [62]. Everyone uses different coping strategies when faced with a stressor [63]. Problem-focused strategies are strategies such as planning, active coping, seeking instrumental support, and positive reframing that will be implemented when faced with stress. Emotion-focused strategies will use methods such as self-distraction, substance use, behavioral disengagement, and denial. The avoidant coping strategy uses methods such as self-distraction, substance use, behavioral disengagement, and denial [64]. By adopting the avoidant coping strategy, firefighters can avoid the stress derived from the COVID-19 pandemic. However, to truly solve the problem, past studies have confirmed that the strategies to be adopted are problem-focused strategies [65]. However, the higher the feeling of COVID-19 stress, the less willing the firefighters are to adopt problem-focused strategies.

The social instability caused by the pandemic has drawn attention to stress coping strategies [66,67]. Stress coping strategies are behavioral efforts to reduce the negative effects of stressful situations [68]. The use of different coping strategies during the pandemic to address stressors is the focus of the discussion [69]. Some studies have revealed that most people adopt problem-focused coping strategies when they feel unsafe during the pandemic. For firefighters, in the face of public health emergencies, they must also adopt problem-focused strategies.

Taking the Gulf War as an example, positive coping strategies are believed to reduce post-traumatic stress disorder (PTSD) [70]. It was also found in the Iraq War in the past that coping strategies adopted by soldiers can reduce PTSD [71].

**H3.**  *Problem-focused strategies negatively affect PTSD.*

**H4.**  *COVID-19 stress negatively affects problem-focused strategies.*

Some studies conducted in the past have discovered that the risk of infection is a stressor for hospital workers [72,73]. Due to the high risk of infection, front-line workers have high work stress and high turnover rate. In the early stage of the outbreak, the number of confirmed cases gradually increased. At that time, personal protective equipment was insufficient, the workload increased, and the risk of infection surged, putting work stress on front-line workers [74,75]. Past studies on COVID-19 indicated that the negative psychological effects of COVID-19 infection last for a long time [76], and finally result in post-traumatic stress disorder.

Yıldırım et al. found that fear of COVID-19 was the cause of personal depression and stress [77]. Rovai et al. found that the fear of COVID-19 is an important factor affecting the ups and downs of human emotion [78]. The COVID-19 outbreak is full of uncertainties, affecting individuals’ mental and physical health. The uncertainty of the pandemic increases the fear of infection for individuals, which gradually increases the psychological fear. A study by Ouellet et al. revealed that people with a low tolerance for uncertainty were more likely to experience stress in the face of the pandemic. The fear includes worries of being infected for an individual and his/her family members. Increased fear of COVID-19 also creates psychological anxiety and stress [79].

Fear of COVID-19, including illness, infection, and death, has a negative psychological impact. In the early stage of the COVID-19 outbreak, many studies focused on the development of drugs and vaccines, and paid less attention to the psychological and social aspects [80]. The pandemic has made people worry not only about the infection of themselves, but also the infection of their family members and friends. Front-line workers have a higher fear of COVID-19 and are more prone to stress [81]. Anxiety is more likely to develop when their family members or relatives are infected with COVID-19 [82]. Firefighters worry that they might become infected when they are on duty, and then spread the virus to their family members, which increases their stress from COVID-19.

Many front-line workers have been found to have the fear of infection [83], which in turn causes stress and anxiety [84]. The psychological problems caused by COVID-19 include increased work stress, as well as worries about the infection of oneself and one’s family members [85]. COVID-19 is a virus that spreads rapidly and has a high mortality rate for the elderly and those with chronic diseases [86]. They are afraid of being infected and of coming into contact with someone who has COVID-19.

Fear of COVID-19 is widespread, causing concern even in areas with low confirmed COVID-19 cases [87]. Sources of traumatic stress due to COVID-19 include infection, fear of death, and changes in family as well as social life [33,43,44]. Therefore, the following hypotheses are made:

**H5.**  *Fear of family infection positively affects COVID-19 stress.*

**H6.**  *Fear of self-infection positively affects COVID-19 stress.*

In countries with formalist oriented culture, there is no common value between administrative levels, resulting in an inconsistency between government and social values [88]. During the COVID-19 pandemic, the inconsistency between civil servants and social values will make the implementation of epidemic prevention policies difficult. Thompson pointed out that there are more generalists in developing countries. They pay more attention to hierarchy and procedures, and fail to notice that they are of purely instrumental origin [40,89,90]. The policies of many developing countries are often fully formulated, but cannot be truly implemented. The pandemic, the spread of the virus and the sources of infection are specialized issues, which cannot be understood and handled by generalists.

A bureaucracy with high formalism is full of pathological behaviors, including lack of authorization, excessive emphasis on control, lengthy official documents, indifference, and fear of innovation. Due to the lack of communication, civil servants cannot make decisions flexibly, making individuals feel a lack of security [91]. During the pandemic, everything is very critical. Firefighters must face people who are infected with COVID-19 and cannot wait for orders all the time, which will create COVID-19 stress.

The administration of developing countries is full of “irrational management” [42]. Since there is no authorization, many small matters have to be decided by the supervisor. The administrative procedures in developing countries are very complicated. On one hand, they are required to administer in accordance with the laws, but on the other hand they do not care about the spirit of the law [42]. The formalism of these legal procedures is considered a part of the self-protection of civil servants. Civil servants mainly care about their own interests, rather than public interests [42].

Civil servants with a high awareness of formalism are less sympathetic to the public [42]. The entire bureaucratic system is full of formal and legalistic structure, and civil servants are unwilling to make risky decisions [42]. In addition, the capriciousness of political leadership makes civil servants lack self-efficacy and a sense of commitment [42]. Regarding the risk of the pandemic, when firefighters lack self-efficiency and a sense of commitment, their COVID-19 stress will also be increased.

**H7.**  *Formalism positively affects COVID-19 stress.*

**Figure 1:**
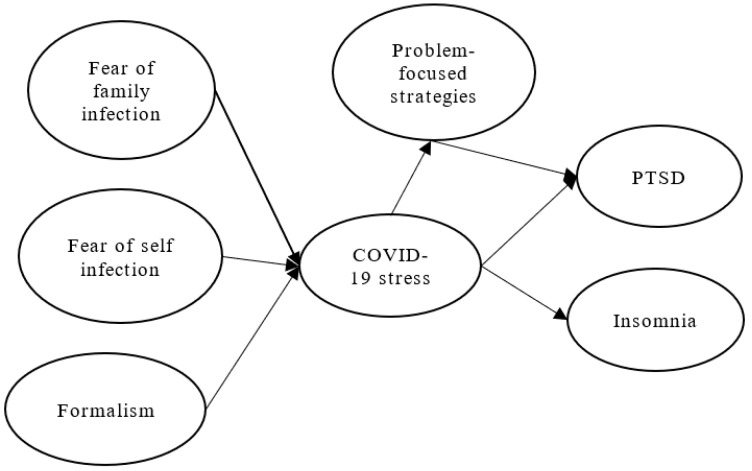
Conceptual framework.

This study argues that firefighters’ fear of infection for themselves and their family increases their COVID-19 stress (see Figure 1). Firefighters’ formalistic cognition of epidemic prevention policies will also increase their COVID-19 stress. The COVID-19 stress of firefighters can further cause PTSD and insomnia problems. Problem-focused strategies adopted by firefighters will reduce PTSD.

## 3. Materials and Methods

### 3.1. Sample, Tools, and Procedure

The total number of firefighters in Taiwan is about 16,313. Firefighters in Taiwan were used as the research object, and 453 valid samples were obtained through convenience sampling. We use G*Power 3.1.9.7 version, set α err prob = 0.05; Power (1-β err prob) = 0.95, and calculate the required samples to be 146. The 453 samples collected in this study exceeded the minimum sample number of 146 calculated by G*Power. Males accounted for 94.0 % of the sample (see Table 1). In terms of age, 20–29 years old accounted for 28.9%; 30–39 years old accounted for 41.9%; 40–49 years old accounted for 18.3%; 50 years old or older accounted for 10.8%. In terms of academic qualifications, junior college accounts for 43.7%; college accounts for 40.8%; postgraduate accounts for 15.5%. In terms of working years, 59.8% have less than 11 years, and 40.2% have more than 12 years. In terms of marriage, unmarried firefighters accounted for 42.8% of the samples. In terms of positions, firefighters, sergeant, and station chief in the sample accounted for 76.8%, 13.7%, and 9.5%. A total of 61.4% of the respondents indicated that they had been exposed to patients with COVID-19 while on duty.

### 3.2. Measures

The items for fear of family infection are modified from the scale developed by Mayer et al. [92]. The items for fear of self-infection are modified from the scale developed by Mayer et al. and Ahorsu et al. [14,92]. The items for PTSD were modified from the scale developed by Weathers et al. [93]. Insomnia items are modified from the scale developed by Morin, Belleville, Bélanger, Ivers [94]. The items of problem-focused coping strategies are modified from the scale developed by Zhao et al. [95]. The items for COVID-19 stress are modified from the scale developed by Taylor et al. [96]. According to the definition of formalism and the scale applied in previous research [97,98], this study designs the following items: I don’t think the epidemic prevention regulations will be the same as the actual implementation. I think the laws of epidemic prevention are difficult to be implemented in practice. I think many epidemic prevention systems are not easy to implement. I think there will be differences in the regulations and implementation of epidemic prevention. The Cronbach α values of all constructs ranged from 0.92 to 0.97 (as shown in Table 2), which was higher than the minimum threshold of 0.60 set by Nunnally [99].

### 3.3. Validity and Reliability Analysis

In this study, SEM (Structure Equation Modeling) software was used to test the reliability and validity of construct and items by confirmatory factor analysis (CFA). In terms of model overall fit measures, the SRMR (Standardized Root Mean Square) of the conceptual model of this study is 0.052 (see Table 2), which is slightly higher than the judgment criterion of 0.05, but still within the acceptable range. The GFI is 0.99, which is higher than 0.90. Based on the model comparison fit measures, NNFI is 0.99, NFI is 0.99, CFI is 0.99, IFI is 0.99, and RFI is 0.99. All of them are higher than the judgment criterion of 0.90, indicating that the hypothetical model can be accepted. In terms of model parsimonious fit measures, PNFI is 0.93 and PGFI is 0.86. Both of them are higher than the standard of 0.50. The above results show the suitability of the conceptual model of this study. The fit between the model and the empirical data also confirms the overall construct validity of this study.

Furthermore, in terms of the factor loading λ, the factor loadings of all constructs ranged from 0.71 to 0.95. All of them are higher than 0.5, which is in line with the recommendations of Hair, Anderson, Tatham and Black (>0.5) [100], indicating that the scale of individual items in this study has an acceptable level of reliability. All the item loading T values in this study reached the statistically significant level, and some of them also confirmed the construct validity and convergent validity of the facets of this study.

The composite reliability (CR) of latent facets can measure the consistency of variables within the facets. According to Hair et al., the CR value should be greater than 0.7 [100]. The CR value of the latent variables in this study is between 0.92 and 0.97, all greater than 0.7, indicating that the latent facets of this study have good internal consistency.

The first Item of all variables is fixed at 1, so there is no *Z* value.

In general, the square root of the average variance extracted for an individual construct should be greater than the correlation coefficient between the construct and other constructs in the model, indicating discriminant validity [101]. The following table summarizes the correlation coefficient matrix between various facets, and the diagonal line is the AVE square root of the construct. The square root of construct AVE in this study is between 0.61 and 0.84 (see Table 3), which is greater than the correlation coefficient between any two facets. In addition, AVE is also greater than MSV and ASV, demonstrating the discriminant validity of this study [100].

The correlation matrix in Table 3 shows the preliminary relationship between the two constructs. COVID-19 stress is positively correlated with fear of family infection, fear of self-infection, and formalism, and their correlation coefficients are: 0.63, 0.77, and 0.40. COVID-19 stress is positively correlated with PTSD and insomnia, and their correlation coefficients are: 0.77, 0.73. Problem-focused strategies are negatively correlated with PTSD and COVID-19 stress, and their correlation coefficients are: −0.13, −0.05.

### 3.4. Controlling for Common Method Variance (CMV)

Common method variance (CMV) is regarded as a variation due to a measurement method that causes an internal consistency error [102,103]. The questionnaires in this study are all self-administered, and common method variance questions may arise. In order to prevent the research results from being affected by common method variance, the questionnaire survey of this paper was conducted in an anonymous manner, and the Likert 5–7 point scale was used in conjunction to reduce the effect of common method variance [102]. The questionnaire design is also based on a fixed operation process, and the item design is based on the principle of simplicity and easy understanding. Items that confuse the participants, are not easy to interpret, or are difficult to answer have been avoided as much as possible.

In terms of post hoc analysis, this study adopted Harman’s one-factor test. In exploratory factor analysis, the unrotated first principal component explained only 42.2%, which is not too high, suggesting that the problem of common method variance in this study is not serious.

## 4. Results

In this study, the path coefficient analysis in structural equation modeling (SEM) is used to test the established hypotheses [104]. It can be seen from Table 4 that the COVID-19 stress of firefighters positively affects PTSD, and the path coefficient is 0.76 (see Table 4), validating H1. This implies that the greater the COVID-19 stress of firefighters, the more severe their PTSD will be.

The COVID-19 stress of firefighters positively affects insomnia with a path coefficient of 0.71, validating H2. This suggests that the greater the COVID-19 stress of firefighters, the more severe their insomnia will be. The path coefficient for the adoption of problem-focused strategies by firefighters which negatively reduces PTSD is −0.08, validating H3. When firefighters adopt problem-focused strategies, their PTSD is reduced.

The path coefficient for the COVID-19 stress of firefighters which negatively affects the adoption of problem-focused strategies is −0.13, validating H4. In the face of COVID-19 stress, firefighters are less willing to adopt problem-focused strategies. The fear of family infection for firefighters positively affects their COVID-19 stress, with a path coefficient of 0.09, validating H5. The firefighters’ fear of self-infection positively affects their COVID-19 stress, with a path coefficient of 0.68, validating H6. Firefighters’ fear of COVID-19 infection of themselves and their family members intensifies their COVID-19 stress.

The formalism of firefighters positively affects COVID-19 stress, with a path coefficient of 0.16, validating H7. When firefighters have a high sense of formalism, they tend to feel that the epidemic prevention regulations are inconsistent with the facts, which increases their COVID-19 stress.

## 5. Discussion

This study found that COVID-19 stress positively affects PTSD among firefighters. Such results are similar to previous research findings [50,51,54]. COVID-19 stress among firefighters can further contribute to anxiety and PTSD. COVID-19 stress has caused changes in the daily life of firefighters and mistrust of supervisors, which can easily lead to PTSD. Firefighters have witnessed many injuries and deaths during the pandemic and are prone to post-traumatic syndrome.

The COVID-19 stress of firefighters is affecting their insomnia. This is similar to the research results of Xu et al. and Zhang et al. [60,61]. The uncertainty of how COVID-19 spreads and the lack of officially approved medicines are causing them stress and insomnia.

This study found that COVID-19 stress among firefighters negatively affects their adoption of problem-focused strategies. The strategies that firefighters can adopt include emotion-focused strategy, avoidant coping strategy and problem-focused strategy. Faced with the pressure of COVID-19 pandemic duty, firefighters are expected to adopt a problem-focused strategy [65]. This study found that the higher the COVID-19 stress, the less willing firefighters are to adopt a problem-focused strategy. When firefighters are willing to adopt a problem-focused strategy to face stress, it will reduce the PTSD of firefighters. This confirmation is similar to the findings of Creech et al. [71].

Fear of contagion to self and family members are both stressors of COVID-19. Firefighters are the front lines transporting COVID-19 patients. In the early stage of the pandemic expansion, there was no clear understanding of the COVID-19 virus and lack of protective equipment. At that time, the pandemic environment made firefighters worry about infection of themselves and their family members, which in turn caused firefighters to develop COVID-19 stress [43,44,45].

This study confirms that formalism cognition of firefighters will increase COVID-19 stress. During the COVID-19 outbreak, the government will enact many executive orders to prevent the spread of the pandemic. When firefighters feel inconsistent with pandemic prevention regulations and implementation, their COVID-19 stress will increase. As Pye said, a bureaucratic system with high formalism lacks communication, flexibility, and decision-making power [91], which makes front-line workers feel more stressed. The volatility of political appointees’ orders during the pandemic will also increase the sense of insecurity and stress among firefighters [42].

## 6. Conclusions

In terms of theoretical implications, this study confirms that formalism does affect firefighters’ COVID-19 stress perception. Most of the formalism literature on administrative management in the past is classified as qualitative research or descriptive statistical analysis. This study is one of the few that applies the concept of formalism to administrative management with inferential statistical analysis. The impact of formalism on the psychological aspects of civil servants has not been thoroughly explored and verified. This study can bridge the theoretical and empirical research gap of formalism.

To avoid the firefighters’ increased fear of COVID-19, they can continue to contact relatives and friends, or take the initiative to seek the assistance of professional psychological counseling and express his/her fears about COVID-19. Firefighters can seek support from supervisors or colleagues to reduce COVID-19 stress on the job. Supervisors have the authority to make decisions about assignments and transfers of duties. During the epidemic, there was a lot of fake news and disinformation spreading. Firefighters seeking COVID-19 information from trusted media can reduce their fear and stress. Mindful practice is a tool for the self-assessment of physical health, mental health, and emotion. Firefighters can also use mindful practice to reduce negative emotions and focus on existing work. Encourage and train firefighters to adopt problem-focused strategies to cope with stress. The reduction of COVID-19 stress in firefighters will reduce their PTSD and insomnia.

## 7. Limitations

The sample of 453 firefighters obtained in this study is not very small. However, it is still hoped that future related research can increase the sample size and supplement the discussion of qualitative data. The sample size of a single study is not sufficient to draw conclusions about firefighters in all countries. This study adopts a self-reported questionnaire, and biases of selective memory, telescoping effect, attribution, and exaggeration may occur. Due to limited research time and financial resources, this study can only take a cross-sectional research approach. It is suggested that future research can be explored in depth in the form of longitudinal studies. This study only collects samples of firefighters in Taiwan, and there may be cultural bias. It is suggested that future research can collect multi-country samples to facilitate the generalization of research results to more countries.

## Figures and Tables

**Table 1:** Sample basic information.

	Counts	Percentage (%)		Counts	Percentage (%)
Gender			Seniority		
Male	426	94.0 %	1 to 3 years	115	25.4 %
Female	27	6.0 %	4 to 7 years	98	21.6 %
Age			8 to 11 years	58	12.8 %
20–29 years old	131	28.9 %	12 to 15 years	59	13.0 %
30–39 years old	190	41.9 %	16 years or more	123	27.2 %
40–49 years old	83	18.3 %	Marriage		
50 years old or older	49	10.8 %	Unmarried	194	42.8 %
Education level			Married	253	55.8 %
Junior college	198	43.7 %	Other	6	1.3 %
College	185	40.8 %	Position		
Postgraduate	70	15.5 %	Firefighters	348	76.8 %
			Sergeant	62	13.7 %
			Station Chief	43	9.5 %

**Table 2:** Item loading and model fits.

Variables	Items	Lambda	*Z* Value	Composite Reliability	Cronbach’s Alpha
Fear of family infection	FFI 1	0.90		0.97	0.97
	FFI 2	0.93	172.5		
	FFI 3	0.94	173		
	FFI 4	0.89	170.2		
	FFI 5	0.90	164.2		
	FFI 6	0.91	166.5		
	FFI 7	0.95	170.7		
Fear of self-infection	FSI 1	0.88		0.94	0.94
	FSI 2	0.86	188.6		
	FSI 3	0.87	185.4		
	FSI 4	0.94	191.6		
	FSI 5	0.81	177.6		
	FSI 6	0.78	175.2		
Formalism	FOR 1	0.88		0.93	0.93
	FOR 2	0.88	73		
	FOR 3	0.91	74.7		
	FOR 4	0.83	71		
PTSD	PTSD 1	0.80		0.92	0.92
	PTSD 2	0.79	169		
	PTSD 3	0.84	173.2		
	PTSD 4	0.71	165		
	PTSD 5	0.72	172		
	PTSD 6	0.78	161.7		
	PTSD 7	0.82	173.7		
Insomnia	IN 1	0.86		0.93	0.92
	IN 2	0.72	159.9		
	IN 3	0.81	168.3		
	IN 4	0.78	170.1		
	IN 5	0.81	161.4		
	IN 6	0.84	177.5		
	IN 7	0.79	164.5		
Problem-focused strategies	PFS 1	0.81		0.95	0.95
	PFS 2	0.80	43.9		
	PFS 3	0.86	44.3		
	PFS 4	0.91	45.5		
	PFS 5	0.93	45.2		
	PFS 6	0.89	45		
	PFS 7	0.75	42.2		
COVID-19 stress	COS 1	0.81		0.92	0.94
	COS 2	0.79	164.7		
	COS 3	0.86	175.6		
	COS 4	0.74	156		
	COS 5	0.75	161.3		
	COS 6	0.85	171.4		
	COS 7	0.74	157		

Note: FFI = Fear of family infection; FSI = Fear of self-infection; FOR = Formalism; IN = Insomnia; PTSD = Post-traumatic stress disorder; PFS = Problem-focused strategies; COS = COVID-19 stress.

**Table 3:** Square root of AVE and inter-correlations.

	FFI	FSI	FOR	PTSD	IN	PFS	COS	ASV	MSV	AVE
FFI	(0.92)							0.28	0.55	0.84
FSI	0.74	(0.86)						0.38	0.59	0.73
FOR	0.34	0.32	(0.88)					0.09	0.16	0.77
PTSD	0.55	0.76	0.30	(0.78)				0.36	0.60	0.61
IN	0.55	0.66	0.27	0.77	(0.80)			0.32	0.59	0.64
PFS	0.04	0.03	0.16	−0.13	−0.07	(0.85)		0.01	0.03	0.73
COS	0.63	0.77	0.40	0.77	0.73	−0.05	(0.80)	0.38	0.60	0.63

Note: The figures in parentheses indicate the square root of AVE of the study constructs. MSV = maximum share variance; ASV = average share variance. FFI = Fear of family infection; FSI = Fear of self-infection; FOR = Formalism; IN = Insomnia; PTSD = Post-traumatic stress disorder; PFS = Problem-focused strategies; COS = COVID-19 stress.

**Table 4:** Path coefficients (coefficients, STDEV, *Z*-values).

Hypothesis	Causal Path			Path Coefficient	Standard Deviation	*Z* Value
H1	COVID-19 stress	→	PTSD	0.76 **	0.03	24.91
H2	COVID-19 stress	→	Insomnia	0.71 **	0.04	21.66
H3	Problem-focused strategies	→	PTSD	−0.08 **	0.04	−2.87
H4	COVID-19 stress	→	Problem-focused strategies	−0.13 **	0.04	−2.82
H5	Fear of family infection	→	COVID-19 stress	0.09 *	0.04	2.03
H6	Fear of self-infection	→	COVID-19 stress	0.68 **	0.04	15.28
H7	Formalism	→	COVID-19 stress	0.16 **	0.06	5.41

Note: *, and ** represent statistical significance at *p* < 0.05, and *p* < 0.01, respectively.

## Data Availability

Not applicable.
